# Physicians’ Perceptions of a Situation Awareness–Oriented Visualization Technology for Viscoelastic Blood Coagulation Management (Visual Clot): Mixed Methods Study

**DOI:** 10.2196/19036

**Published:** 2020-12-04

**Authors:** Tadzio Raoul Roche, Sadiq Said, Julian Rössler, Malgorzata Gozdzik, Patrick Meybohm, Kai Zacharowski, Donat R Spahn, Christoph B Nöthiger, David W Tscholl

**Affiliations:** 1 Institute of Anesthesiology University of Zurich and University Hospital Zurich Zürich Switzerland; 2 Department of Anesthesiology University Hospital Würzburg Würzburg Germany; 3 Department of Anesthesiology, Intensive Care Medicine and Pain Therapy University Hospital Frankfurt Frankfurt am Main Germany

**Keywords:** blood coagulation, hemostasis, blood coagulation test, point of care, rotational thromboelastometry, Visual Clot, decision making, survey and questionnaires, situation awareness, user-centered design, qualitative research, visualization, avatar

## Abstract

**Background:**

Viscoelastic tests enable a time-efficient analysis of coagulation properties. An important limitation of viscoelastic tests is the complicated presentation of their results in the form of abstract graphs with a multitude of numbers. We developed Visual Clot to simplify the interpretation of presented clotting information. This visualization technology applies user-centered design principles to create an animated model of a blood clot during the hemostatic cascade. In a previous simulation study, we found Visual Clot to double diagnostic accuracy, reduce time to decision making and perceived workload, and improve care providers’ confidence.

**Objective:**

This study aimed to investigate the opinions of physicians on Visual Clot technology. It further aimed to assess its strengths, limitations, and clinical applicability as a support tool for coagulation management.

**Methods:**

This was a researcher-initiated, international, double-center, mixed qualitative-quantitative study that included the anesthesiologists and intensive care physicians who participated in the previous Visual Clot study. After the participants solved six coagulation scenarios using Visual Clot, we questioned them about the perceived pros and cons of this new tool. Employing qualitative research methods, we identified recurring answer patterns, and derived major topics and subthemes through inductive coding. Based on them, we defined six statements. The study participants later rated their agreement to these statements on five-point Likert scales in an online survey, which represented the quantitative part of this study.

**Results:**

A total of 60 physicians participated in the primary Visual Clot study. Among these, 36 gave an interview and 42 completed the online survey. In total, eight different major topics were derived from the interview field note responses. The three most common topics were “positive design features” (29/36, 81%), “facilitates decision making” (17/36, 47%), and “quantification not made” (17/36, 47%). In the online survey, 93% (39/42) agreed to the statement that Visual Clot is intuitive and easy to learn. Moreover, 90% (38/42) of the participants agreed that they would like the standard result and Visual Clot displayed on the screen side by side. Furthermore, 86% (36/42) indicated that Visual Clot allows them to deal with complex coagulation situations more quickly.

**Conclusions:**

A group of anesthesia and intensive care physicians from two university hospitals in central Europe considered Visual Clot technology to be intuitive, easy to learn, and useful for decision making in situations of active bleeding. From the responses of these possible future users, Visual Clot appears to constitute an efficient and well-accepted way to streamline the decision-making process in viscoelastic test–based coagulation management.

## Introduction

For optimal perioperative bleeding management, a quick and reliable assessment of the patient’s blood coagulation function is of utmost importance [1-3]. As laboratory coagulation testing can take more than an hour [4] to produce results, viscoelastic point-of-care devices, such as rotational thromboelastometry (ROTEM, Instrumentation Laboratory/Werfen) and thromboelastography (Haemonetics) play an important role in guiding hemostatic interventions in a time-efficient manner [5]. Previous studies showed that the utilization of viscoelastic testing can improve the patient outcome in those with bleeding trauma [3,6-9]. Hence, the European guidelines on trauma management recommend viscoelastic testing to enable goal-directed bleeding management [10]. Furthermore, viscoelastic point-of-care coagulation testing reduces the transfusion of allogeneic blood products in trauma [2,10]. Previous studies also showed the benefits of its use in cardiac [1,11,12], transplant [13], neuro [14], and pediatric surgery [5].

However, the abstract presentation of viscoelastic test results complicates its handling in clinical routine. This in turn shows the demand for a tool that facilitates the interpretation of viscoelastic readings. To serve this apparent need, Visual Clot technology [15] was developed. Visual Clot is an alternative situation awareness–oriented visualization technology created by adhering to the principles of user-centered design [16]. Instead of abstract shapes and a multitude of numbers, as in the conventional presentation of the ROTEM results, Visual Clot technology displays a three-dimensional animated model of a blood clot that corresponds to the real phenomena one would see when looking at the blood clot through an electron microscope. In an international dual-center study [15], Visual Clot technology enabled the participating physicians to make twice as many correct diagnoses quicker and with improved diagnostic confidence and reduced perceived cognitive workload compared with standard viscoelastic test results.

This study aimed to explore and capture the opinions of the 60 anesthesiologists and intensive care specialists from the previous Visual Clot study [15] on this new visualization technology. The results will help define the strengths and limitations of this visual decision aid. Further, this study examined the applicability of Visual Clot as an additional tool for coagulation management from the viewpoint of its potential future users.

## Methods

### Approval and Consent

The leading ethics committee (the Cantonal Ethics Committee of the Canton of Zurich in Switzerland) reviewed the study protocol and issued a declaration of no objection (Business Management System for Ethics Committees Number 2018-00933). We obtained written informed consent from each participant for the use of their data.

### Study Design

We conducted this study at the University Hospital Frankfurt (UKF) in Germany and the University Hospital Zurich (USZ) in Switzerland. We included anesthesiologists and intensive care physicians from these two hospitals. Both study centers routinely use ROTEM-guided hemostatic resuscitation.

This study is a researcher-initiated, international, dual-center, mixed qualitative-quantitative study about the opinions of anesthesia and intensive care physicians regarding Visual Clot technology. The methods employed included interviewing physicians after their first contact with Visual Clot technology and jointly generating field notes based on their answers. Further, we created statements derived from these field note responses and asked the same group of physicians to rate the statements in an online survey.

### Previous Visual Clot Study and Participant Interviews

**Figure 1:**
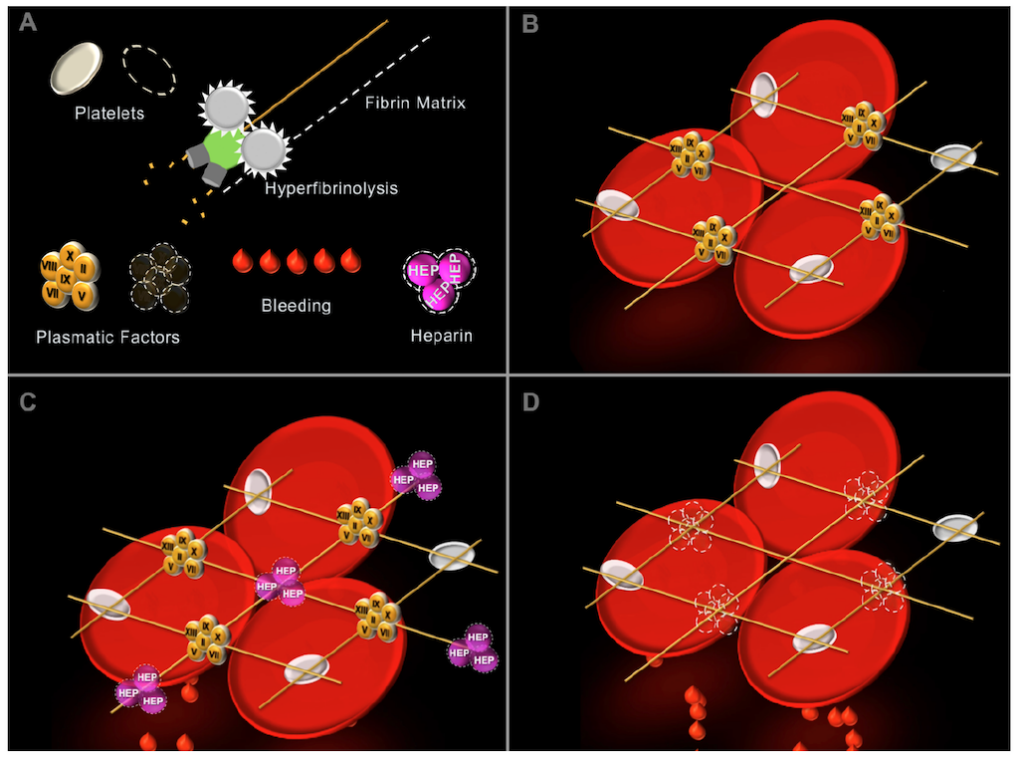
Graphics illustrating Visual Clot technology. A: The different coagulation components that Visual Clot represents as either present or absent (when dashed). B: A healthy Visual Clot with all coagulation elements present in sufficient quantity. C: A bleeding Visual Clot with heparin effect. D: A bleeding Visual Clot indicating the absence of plasmatic factors.

We held interviews for the qualitative part of this study at the end of the data collection sessions of the previously published Visual Clot study. In the mentioned study, the participating physicians evaluated 12 coagulation scenarios in randomized order. They solved each scenario twice, once only observing Visual Clot and once only using the conventional viscoelastic test results. Before the testing took place, the participants received brief individual training and subsequently solved six scenarios using Visual Clot to become familiar with its features. We based the individual training on a Microsoft PowerPoint presentation (Microsoft Corporation). This aimed to explain all visualizations of Visual Clot and enabled the participants to ask questions in case of uncertainties. Multimedia Appendix 1 shows the training video for the current version of Visual Clot. Figure 1 explains and illustrates some visualizations of Visual Clot technology.

In an undisturbed environment, we asked the physicians about the advantages and disadvantages of Visual Clot. There was no further guidance by the investigators, who all had previous experience with qualitative research. While the physicians were encouraged to freely verbalize their thoughts, the data collector simultaneously took field notes in a Microsoft Word (Microsoft Corporation) document. After the interview session, the field notes were presented to the participants. They were asked whether they agreed with them and were encouraged to determine final adjustments and changes.

### Qualitative Analysis

**Figure 2:**
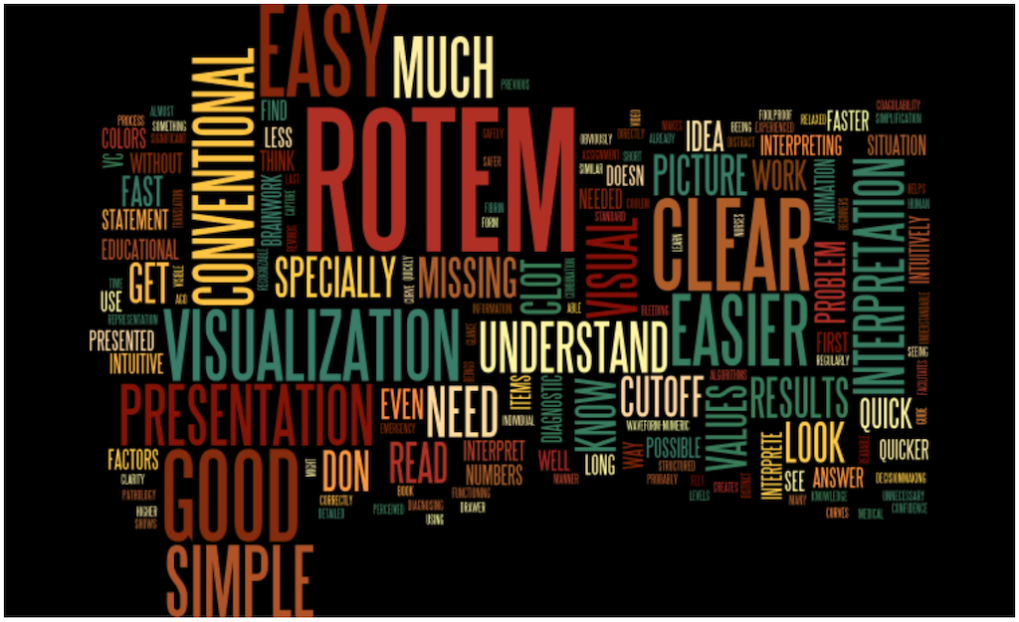
A tag cloud as a quantitative graphic illustration of the most common words in the interview field notes. This word cloud was created using Wordle.net. Common English words (the, and, to, etc) are not displayed. Frequently occurring terms have larger font sizes.

**Figure 3:**
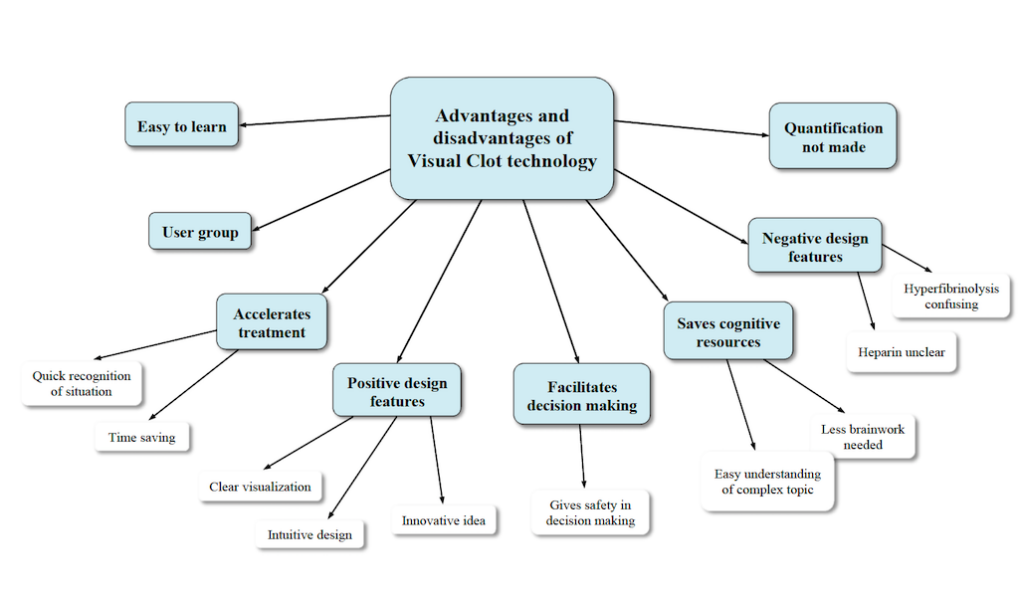
The coding template displaying the major topics and associated subthemes. We generated this through deductive coding via word count and inductive free coding of recurring topics in the interview field notes. A total of 36 participants were interviewed.

We used a template approach in the qualitative analysis [17]. This involves grouping topics using a coding template. The topics are often predefined in advance. During further data analysis, new topics are added or existing ones are revised [17]. First, we translated the original answers from German to English using DeepL [18]. All translated field notes are available in Multimedia Appendix 2. After translation, we used Microsoft Word to identify the most common words and created a tag cloud (Figure 2) as its quantitative graphic illustration using Wordle [19]. We ignored common English words (the, and, to, etc) and unified word groups with the same word root. The two physicians and study authors TRR and DWT were both involved in the qualitative analysis of the field notes. TRR is a resident anesthesiologist who did not partake in the interviewing process. DWT is a senior anesthesiologist with previous experience in qualitative and patient safety research. Adhering to the criteria for reporting qualitative research [20], TRR and DWT developed a coding template as a rating system, using both word count and inductive coding based on recurring topics in the field note responses. We modified the initial coding template by successive reading, coding of the data, and discussions with other authors. After multiple data coding events, we agreed upon a final template. The two study authors mentioned above then independently rated all field notes using an illustrated final coding template, which has been provided in Figure 3. Interrater reliability was calculated to investigate the consistency of the implementation of the rating system. In cases of disagreement between these two examiners, a final code for the respective field note response was jointly determined.

### Quantitative Analysis

Literature provides several arguments why qualitative data could be combined with a quantitative analysis. One of the reasons for this is that quantitative data can help to generalize and confirm specific observations made in a qualitative analysis [21]. To further examine the qualitative research part of this study and Visual Clot applicability, we performed a quantitative assessment of the derived statements using a web-based online survey. We defined six partially generalized statements and asked the participants to rate them on five-point Likert scales with responses ranging from “strongly disagree” to “strongly agree.” Out of all six statements, four directly refer to the previously identified major topics. We specifically added two additional statements to the questioning that we considered necessary. These two aimed to obtain a deeper understanding of Visual Clot technology applicability. Using SurveyMonkey (SVMK Inc), we created the open online survey and tested its usability before sending the study link via email to all physicians who participated in the previous Visual Clot study and still worked at the respective institutions. We informed the participants that the survey takes about 1 minute to fill out. Participation was voluntary, no incentives were offered, and no personal identifying information was collected. The participants were able to review and change their answers before completion. A single reminder to complete the survey was sent after 2 weeks. Multimedia Appendix 3 displays the translated wording of the survey invitation announcement as well as the reminder mail. We completed the data collection 1 week after the reminder period expired.

### Statistical Analysis

We provide the quantitative analysis data of the online survey as median and interquartile range for all statements. Using the Wilcoxon signed-rank test, we evaluated the difference between the median for each of the statements and the neutral answer. We considered *P*<.05 to indicate statistical significance. The qualitative part of this study aimed to identify the pros and cons using the new visualization technology. With the evaluated statements of the online survey, we aimed to further quantify the agreement or disagreement. This procedure additionally increases the relevance of the statements made.

### Data Sharing Statement

The translated interview field notes are available in Multimedia Appendix 2. We report all other data in this manuscript.

## Results

### Study and Participant Characteristics

This study included the same participants as in the previous Visual Clot study [15]. A total of 60 anesthesia and intensive care physicians participated. Half of them were working at the USZ and the other half at the UKF. After collecting data from the first 24 participants of the Visual Clot study, we decided to conduct systematic interviews as we found that the participants provided very informative feedback on the technology. We interviewed all of the participants at the UKF and six participants at the USZ (total of 36 out of 60 [60%]). Regarding the online survey, we present the results according to the checklist for reporting results of internet e-surveys [22]. We sent out the study link as an invitation to all physicians who still worked at the respective institutions. We checked the IP address displayed on the SurveyMonkey website to identify potential duplicate entries from the same user. Since the computers in the hospitals are shared and the workspace usually only changes daily, we considered a difference of at least 24 hours as appropriate for entries from the same IP address. Of all invitations sent out for the online survey, the participation rate was 86% (42/49). Table 1 further outlines the study and participant characteristics in detail.

**Table 1:** Study and participant characteristics.

Characteristics		Value
**Study characteristics**		
	Total number of participants, n	60
	Total number of interviewed participants (field notes) (N=60), n (%)	36 (60)
	Online survey participation rate (N=49), n (%)	42 (86)
	Online survey completion rate (N=42), n (%)	42 (100)
**Participant characteristics**		
	Female sex (N=60), n (%)	23 (38)
	Senior physicians (N=60), n (%)	35 (58)
	Resident physicians (N=60), n (%)	25 (42)
	Anesthesia experience in years, mean (IQR)	8 (4-11)
	Number of annually interpreted ROTEM^a^ readings per physician, mean (IQR)	40 (10-53)

^a^ROTEM: rotational thromboelastometry.

### Part I: Qualitative Analysis of Interview Answers

The analysis of word count showed the following 12 most frequently occurring words or word groups in the participants’ field note responses: visual/visualization/visually (24/36 participants, 67%), easy/easier/easily (18/36 participants, 50%), ROTEM (18/36 participants, 50%), interpretation/interpret/interpreting (13/36 participants, 36%), presentation (12/36 participants, 33%), good (11/36 participants, 31%), clear (11/36 participants, 31%), simple/simplification/simplicity (10/36 participants, 28%), understand/understood/understandable (10/36 participants, 28%), quick/quicker/quickly (6/36 participants, 17%), cut-off/cut-off values (6/36 participants, 17%), and hyperfibrinolysis (6/36 participants, 17%). Figure 2 provides the tag cloud created from the word count of the field note responses.

Through inductive free coding, we identified eight major topics with associated subthemes. Figure 3 displays the generated coding template, which was used as a coding system. The two study authors TRR and DWT rated all 36 field note responses independently. A total of 131 codes were assessed. Interrater reliability was 71%, with a Cohen kappa of 0.665. After the first run, TRR and DWT discussed all differences and agreed on a coding in the case of disagreement. The second run showed an interrater agreement of 100% between the coding of TRR and DWT. Table 2 outlines all major topics with participant counts, percentages, and examples.

**Table 2:** The major topics with participant count, percentages, and examples (N=36)

Major topic	Examples
Positive design features (29 participants, 81%)	Participant #15: Intuitive kind of presentation. Participant #7: Clear presentation, no unnecessary information to distract. Participant #1: Obviously, something like this was missing.
Facilitates decision making (17 participants, 47%)	Participant #19: Creates clarity in emergency situations. Participant #30: Perceived diagnostic confidence is higher.
Saves cognitive resources (15 participants, 42%)	Participant #9: Don’t have to think as much as with conventional ROTEM. Participant #16: Couldn’t imagine, that it could be presented in such a simple way!
Easy to learn (14 participants, 39%)	Participant #05: Even without extensive previous education. Participant #33: Easy. Self-explaining. Participant #13: Fast learning.
Accelerates treatment (13 participants, 36%)	Participant #19: You can see immediately where the problem is. Participant #24: Quickly realize what the problem is. Participant #26: Faster with the Visual Clot. Participant #30: You get the answer much faster.
User groups (9 participants, 25%)	Participant #25: Can be used safely by all educational levels.
Quantification not made (17 participants, 47%)	Participant #14: No quantitative data or information.
Negative design features (17 participants, 47%)	Participant #22: Missing fibrin is not detected and understood easily. Participant #23: Heparin effect is a little tricky to interpret.

### Themes

#### Positive Design Features

Of the 36 participants, 29 (81%) made comments that fit the major topic positive design features. After additional inductive free coding, this topic was further divided into the three subthemes “intuitive design,” “clear visualization,” and “innovative idea.” Some participants perceived the visualization as intuitive, while others pointed out its clear visualization of standard ROTEM results. Participant #7 stated that there was no unnecessary information to distract. Others indicated Visual Clot as innovative, as they perceived this idea to be missing.

#### Facilitates Decision Making

Of the 36 participants, 17 (47%) noticed during the interview process how Visual Clot technology facilitated their decision making on fulfilling the given task. After free inductive coding, this major topic was broken down into the subtheme “safety in decision making” as some of these participants mentioned this aspect. Participant #2 stated how using Visual Clot made him less afraid of getting involved with the ROTEM analysis. Additionally, participant #19 pointed out that the simplicity of Visual Clot technology created clarity in emergency situations.

#### Saves Cognitive Resources

Of the 36 participants, 15 (42%) indicated that using Visual Clot, the user saves cognitive resources compared with standard ROTEM readings. The two subthemes “less brainwork needed” and “easy understanding of complex topic” are derived from this major topic. Some participants mentioned that they did not have to think as much using Visual Clot. Another participant reasoned that using Visual Clot, he did not need to memorize the cut-off values.

#### Easy to Learn

Of the 36 participants, 14 (39%) remarked that Visual Clot technology is easy to learn. Participant #25 felt that after a short instruction, one is already able to use Visual Clot correctly.

#### Accelerating Treatment

Of the 36 participants, 13 (36%) found Visual Clot to accelerate treatment. This major topic was subdivided into the aspects “quick recognition of situation” and “time saving.” Some participants mentioned that using Visual clot, most of the information is visible at a single look. Further, it helps them to interpret the coagulation status faster.

#### User Groups

Of the 36 participants, 9 (25%) made comments that fit the major topic user groups. All of them shared the opinion that Visual Clot would especially help an inexperienced user in the interpretation of ROTEM readings. Participant #25 noted that the visualization technology can be safely used at all educational levels.

#### Quantification Not Made

Of the 36 participants, 17 (47%) had concerns about the missing quantitative data using Visual Clot. Participant #16 suggested to show the visualization technology and the conventional ROTEM results next to each other to overcome this issue. Some mentioned how Visual Clot does not show the extent of the coagulation disorder. Participant #2 was concerned about whether this technology was reliable.

#### Negative Design Features

Of the 36 participants, 17 (47%) made comments that fit the major topic “negative design features.” Problems in differentiating between hyperfibrinolysis and a low fibrinogen state were mentioned by several participants. Moreover, the heparin effect was difficult to interpret. Participant #1 found the heparin visualization confusing.

### Part II: Quantitative Analysis of Statements Rated in the Online Survey

**Figure 4:**
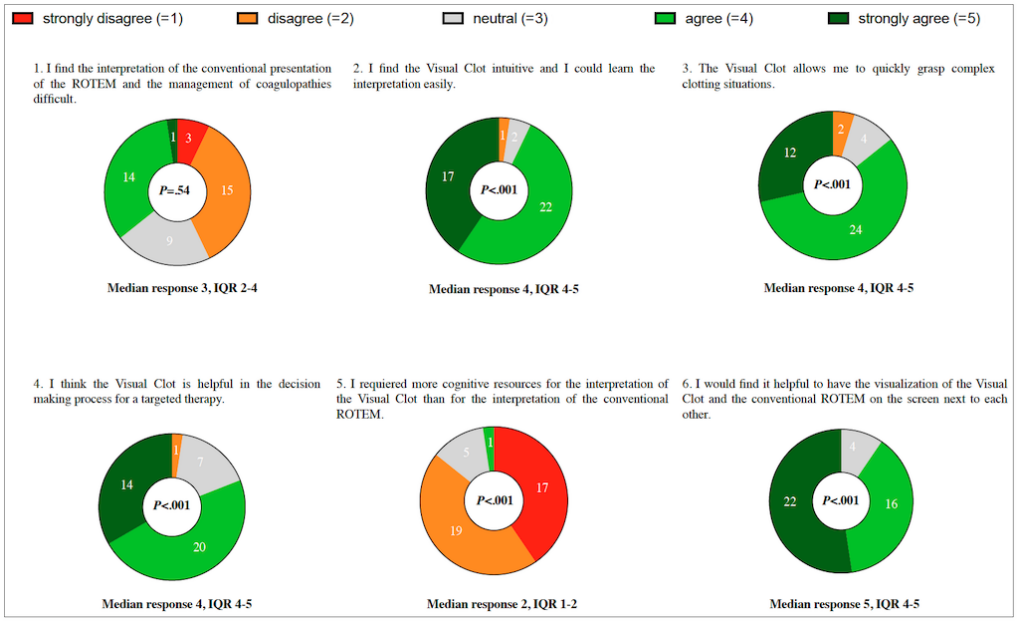
Online survey results presented as donut parts of whole charts with the number of participants who chose a particular category (N=42). We present the results as medians and interquartile ranges and provide *P* values. ROTEM: rotational thromboelastometry.

We illustrate the ratings of the six statements from the online survey in Figure 4. The sample medians of statements two to six all differed statistically significantly from neutral (*P*<.001). The first statement showed that half of the participants considered the use of ROTEM readings in the management of coagulopathies difficult, whereas the other half did not.

The six statements with the evaluation of the online survey rating results are as follows:

(1) “I find the interpretation of the conventional presentation of the ROTEM and the management of coagulopathies difficult.” This statement does not differ statistically significantly from neutral (*P*=.54).

(2) “I find Visual Clot intuitive and I could learn the interpretation easily.” Of the 42 participants, 39 (93%) agreed or strongly agreed with this statement (*P*<.001).

(3) “Visual Clot allows me to quickly grasp complex clotting situations.” Of the 42 participants, 36 (86%) agreed or strongly agreed with this statement (*P*<.001).

(4) “I think Visual Clot is helpful in the decision-making process for a targeted therapy.” Of the 42 participants, 34 (81%) agreed or strongly agreed with this statement (*P*<.001).

(5) “I required more cognitive resources for the interpretation of Visual Clot than for the interpretation of the conventional ROTEM.” Of the 42 participants, 1 (2%) agreed with this statement (*P*<.001).

(6) “I would find it helpful to have the visualization of Visual Clot and the conventional ROTEM on the screen next to each other.” Of the 42 participants, 38 (90%) agreed or strongly agreed with this statement (*P*<.001).

## Discussion

### Principal Findings

This mixed-methods study analyzed the opinions of physicians regarding Visual Clot, a new situation-awareness oriented visualization technology for viscoelastic coagulation management. The main findings were that the participants perceived Visual Clot as intuitive, easy to learn, and helpful in the decision-making process for ROTEM-guided coagulation management. Further, they found that Visual Clot gave them a good overview of the clotting situation. The main criticism concerned its missing quantification. The participants preferred this visualization on a screen next to the conventional ROTEM readings. In an environment where health care providers are confronted with increasingly polymorbid patients and complicated diagnostic tools [23,24], more efficient assistive technologies leading to safer transmission of critical information will be of lasting importance for human performance and patient safety.

Our study showed a high level of physician acceptance and satisfaction with the new tool. Eighty-one percent (34/42) of the participants regarded the animated blood clot as useful in the decision-making process for coagulation management. Only one participant (N=42, 2%) agreed to the statement “I required more cognitive resources for the interpretation of Visual Clot than for the interpretation of conventional ROTEM.” The participant responses corresponded to the reactions to the similarly well-accepted Visual Patient technology [25]. In that assessment, only 11% (4/38) of the questioned subjects considered that technology not helpful in patient monitoring after their first contact with it [26]. Visual Patient is a situation awareness–oriented visualization for patient vital signs with comparable effect size in information transfer improvement as that of Visual Clot [27-30]. Ninety-three percent (39/42) of the participants considered Visual Clot intuitive to interpret and easy to learn. In the Visual Patient study [26], 82% (31/38) of the respondents attributed the same characteristics to Visual Patient. The higher complexity of Visual Patient with far more visualizations may have caused the 10% difference in this aspect between the two technologies. Intuition enables humans to apply a new tool utilizing mainly unconscious processing and previously learned experiential knowledge [31]. Cognitive ease when learning a new technology is crucial for user acceptance [32]. Indeed, previous medical visualization technologies failed because they were too difficult to understand [33,34]. We drafted Visual Clot and its visualizations based on principles of logic, human-computer interaction, and results of prior work in medical interface and user-centered design [16,34]. According to the theory by Wittgenstein [35], a coherent image or model has a meaningful commonality with the reality it is intended to reflect. To achieve this, we designed Visual Clot as a model of a blood clot. This philosophy is in line with results from the study by Wachter et al, which show that an anatomically correct interface is particularly intuitive [33]. A previous National Aeronautics and Space Administration publication outlines the various hierarchical levels of information representation [36]. It highlights the “order of wholeness” achieved by integrating the required information into a single display as the highest level of such presentation. In order to increase the cognitive receptiveness of information, we intentionally animated Visual Clot in a playful way [31,32]. This enhances user accessibility to the otherwise dry data-driven conventional result printouts.

Prior to taking part in the study, none of the participants had ever seen Visual Clot technology. However, the number of correct decisions, time to decision, diagnostic confidence, and workload improved greatly with the new tool compared with conventional ROTEM result presentation [15]. This study reveals high agreement between user perceptions and the function of the technology described in the previous Visual Clot study. This correspondence between expected and provided function is another essential feature for the success of new technologies. If this agreement does not occur, it can lead to false expectations, frustration, and ultimately reduced user acceptance. Most of the answers given concerning its design features were positive (29/36, 81%). Nevertheless, we identified some design features that led to confusion. For example, one visualization aimed to show that a test for heparin was carried out but turned out negative. This visualization intended to show an excluded heparin effect, but it confused some participants. For this reason, we modified the current version of Visual Clot. Now, it only displays heparin as present or not present. This is more in line with its situation awareness–oriented intention to show only essential information about coagulation disorders. Moreover, based on the participants’ feedback, we further refined the visualization of hyperfibrinolysis and fibrin deficiency. We follow both quantitative (ie, participants’ performance with a particular visualization) and qualitative study results in such design modifications. Qualitative feedback is often valuable to understand why a particular visualization is not working. This provides clues on how we can adapt it.

Eighty-six percent (36/42) of the participants agreed to the statement that Visual Clot allowed them to grasp complex coagulation situations more quickly. Ninety percent (38/42) of the participants considered it helpful to have Visual Clot and the conventional ROTEM on the screen next to each other. We regard such an integrated display mode as optimal, as Visual Clot technology converts the continuous numerical values of standard test outputs into categorical visualizations. For example, there can be too few, a healthy amount of, or too many platelets. This simplification has the advantage that information is understood quickly and easily, bearing in mind that this reduces the resolution of given data. Indeed, 47% (17/36) of the interview participants made comments regarding the missing quantification of the disorders using Visual Clot. Hence, an ideal implementation must combine the benefits of Visual Clot, which are fast and easy situation recognition, with the high precision advantage of the conventional method. A hospital may configure the visualization limits of Visual Clot according to the values of the local coagulation algorithm. This could help enforce the local standard procedure.

The main goal of Visual Clot technology is still to simplify complex viscoelastic test results in real time to facilitate the care providers’ overview of the clotting situation. This study showed how potential future users perceived this technology after using it for the first time. Opinions, such as good and clear visualizations and ability to obtain a good overview of the clotting situation in a time-efficient manner, fully support our main intention. Acutely bleeding patients benefit from faster and more accurate diagnosis and treatment. This study made us aware about the desire of our fellow physicians for such technology, from which we can draw fundamental motivation to further develop its concept.

### Limitations

This study has several limitations. The opinions obtained cannot be extrapolated to a larger population because qualitative research does not assess statistical significance. Its only goal is to collect and present the broadest possible range of opinions and views. Second, we questioned the participants after their first contact with the new technology in a controlled instruction setting. Its use in the everyday clinical routine could alter the care providers’ opinions. However, we consider it unlikely that Visual Clot would not be perceived as positively in clinical routine as it was in this study setting, as we observed widespread approval by anesthesiologists and intensive care physicians, who are the potential future users. Nevertheless, further research is needed to identify the strengths and weaknesses of Visual Clot technology in a clinical setting. Another limitation concerns the selection of participants. We did not include participants randomly, but selected them according to their availability in the daily clinical routine. However, the high participation rate in the quantitative analysis reduces potential selection bias. Finally, we conducted this study in university hospitals with high standards of care in central Europe. Users’ opinions may be different in other parts of the world. More research is needed in this respect as well.

### Conclusions

A group of anesthesiologists and intensive care physicians regarded Visual Clot to be intuitive, easy to learn, and useful for the decision-making process in acute clotting disorders. The limitation of the visualization technology resulting from the translation of continuous measurement values into categorical values was the most frequently mentioned potential disadvantage of the technology. A split-screen implementation may be used to combine the advantages of the visualization technology and conventional technology. In this study, Visual Clot appeared to be a well-accepted decision support tool for ROTEM-based coagulation management. Further research is needed to investigate its potential in clinical practice and medical education.

## Appendix

Multimedia Appendix 1 Visual Clot educational video.

Multimedia Appendix 2 Translated field notes of the participant interviews.

Multimedia Appendix 3 Translated online survey announcement.

## Abbreviations

ROTEM: rotational thromboelastometry
UKF: University Hospital Frankfurt
USZ: University Hospital Zurich
