# Supplementary material for: Physicians’ Perceptions of a Situation Awareness–Oriented Visualization Technology for Viscoelastic Blood Coagulation Management (Visual Clot): Mixed Methods Study
Source: JMIR Serious Games. 2020 Dec 4;8(4):e19036. doi: 10.2196/19036 (PMC7748952; doi:10.2196/19036)
Supplement: Multimedia Appendix 2 [file games_v8i4e19036_app2.pdf]

Appendix 2: Translated field notes of the participant interview

Visual Clot – First Contact

Field notes after Visual Clot study participation:

**Participant number**	**(1)**
**Center**	[redacted]
**Gender**	[redacted]
**Job position**	[redacted]
**Anesthesia experience in years in years**	[redacted]
**Performed ROTEM per year**	[redacted]
**Self-rated ROTEM skills**	[redacted]

Question 1: What do you like about Visual Clot? E.g., particular strengths?

Question 2: What do you <u>dislike</u> about Visual Clot? E.g., potential problems, limitations?

Answer question 1:

[Obviously, something like this was missing.] [It's diagnosing at a single look.]

Answer question 2:

Initially, [the Heparin picture was confusing], [but after some explanation, I understood how it works.]

Visual Clot – First Contact

Field notes after Visual Clot study participation:

**Participant number**	**(2)**
**Center**	[redacted]
**Gender**	[redacted]
**Job position**	[redacted]
**Anesthesia experience in years in years**	[redacted]
**Performed ROTEM per year**	[redacted]
**Self-rated ROTEM skills**	[redacted]

Question 1: What do you like about Visual Clot? E.g., particular strengths?

Question 2: What do you dislike about Visual Clot? E.g., potential problems, limitations?

Answer question 1:

[Decision help.] [Easier, faster process.] [Not afraid anymore of getting involved with the ROTEM.]

Answer question 2:

[Is it reliable? Can we trust it?]

Visual Clot – First Contact

Field notes after Visual Clot study participation:

**Participant number**	**(3)**
**Center**	[redacted]
**Gender**	[redacted]
**Job position**	[redacted]
**Anesthesia experience in years in years**	[redacted]
**Performed ROTEM per year**	[redacted]
**Self-rated ROTEM skills**	[redacted]

Question 1: What do you like about Visual Clot? E.g., particular strengths?

Question 2: What do you dislike about Visual Clot? E.g., potential problems, limitations?

Answer question 1:

Animation. [Simplification.] [Standard (normal) and pathology is visible.]

Answer question 2:

[It might not be good for patients with mild coagulation disorders at the limit to normal. There it will be difficult to categorize. Patients with mild coagulation disorders might get overtherapy.]

Visual Clot – First Contact

Field notes after Visual Clot study participation:

**Participant number**	**(4)**
**Center**	[redacted]
**Gender**	[redacted]
**Job position**	[redacted]
**Anesthesia experience in years**	[redacted]
**Performed ROTEM per year**	[redacted]
**Self-rated ROTEM skills**	[redacted]

Question 1: What do you like about Visual Clot? E.g., particular strengths?

Question 2: What do you dislike about Visual Clot? E.g., potential problems, limitations?

Answer question 1:

[You get your answer at a glance.]

Answer question 2:

[Heptem (Heparin effect) unclear.]

Visual Clot – First Contact

Field notes after Visual Clot study participation:

**Participant number**	**(5)**
**Center**	[redacted]
**Gender**	[redacted]
**Job position**	[redacted]
**Anesthesia experience in years**	[redacted]
**Performed ROTEM per year**	[redacted]
**Self-rated ROTEM skills**	[redacted]

Question 1: What do you like about Visual Clot? E.g., particular strengths?

Question 2: What do you dislike about Visual Clot? E.g., potential problems, limitations?

Answer question 1:

[Thanks to this visualization, the interpretation gets easier.] [Specially for visually functioning human beings]

Answer question 2:

[You don't get any (direct) information about which channels have been run.

Function or number of thrombocytes - Multiplate? Do it all, or don't do it -> therapy is often applied gradually and differentiated (for ex. 2 vs 4g Fibr)]

Visual Clot – First Contact

Field notes after Visual Clot study participation:

**Participant number**	**(6)**
**Center**	[redacted]
**Gender**	[redacted]
**Job position**	[redacted]
**Anesthesia experience in years**	[redacted]
**Performed ROTEM per year**	[redacted]
**Self-rated ROTEM skills**	[redacted]

Question 1: What do you <u>like</u> about Visual Clot? E.g., particular strengths?

Question 2: What do you <u>dislike</u> about Visual Clot? E.g., potential problems, limitations?

Answer question 1:

[Cool idea.][Simple and easy visualization.] [Clear presentation of ROTEM results. Attractive visualization.] [To have a legend (explanation) with the picture is important.]

Answer question 2:

[The more experienced may need it less. Primarily, it helps the less experienced.]

Visual Clot – First Contact

Field notes after Visual Clot study participation:

**Participant number**	**(7)**
**Center**	[redacted]
**Gender**	[redacted]
**Job position**	[redacted]
**Anesthesia experience in years**	[redacted]
**Performed ROTEM per year**	[redacted]
**Self-rated ROTEM skills**	[redacted]

Question 1: What do you like about Visual Clot? E.g., particular strengths?

Question 2: What do you dislike about Visual Clot? E.g., potential problems, limitations?

Answer question 1:

[Clear and individual visualization of the results make it possible to directly interprete,] [even without extensive previous education.] [Clear presentation.] [No unnecessary information to distract.]

Answer question 2:

Heparin: The Visual Clot should suggest to make a measurement with Heparinase (HEPTEM), if meaningful and not already done.

[The number of Pac-Man does not correlate to the intensity of hyperfibrinolysis.]

In the beginning, when the Rotem starts, the Visual Clot should give you a hint, that hyperfibrinolysis might be a possible cause of coagulation disorder, even if it cannot yet be detected by ROTEM or Visual Clot. And that therefore reevaluation is needed

Thromobocytes: Same as with hyperfibrinolysis: Needs a hint, that it has to be reevaluated later.

[The Visual Clot gives you no information about the extent of the coagulation disorder. Therefore, you cannot decide about the dose of your treatment.]

Visual Clot – First Contact

Field notes after Visual Clot study participation:

**Participant number**	**(8)**
**Center**	[redacted]
**Gender**	[redacted]
**Job position**	[redacted]
**Anesthesia experience in years**	[redacted]
**Performed ROTEM per year**	[redacted]
**Self-rated ROTEM skills**	[redacted]

Question 1: What do you like about Visual Clot? E.g., particular strengths?

Question 2: What do you dislike about Visual Clot? E.g., potential problems, limitations?

Answer question 1:

[Because there are no numbers you have to read and interpret first,] [you are quicker in interpretation.]

Answer question 2:

[Need to get used to first. Specially when you are experienced in conventional ROTEM.]

[In the Visual Clot, normal amount of fibrin looks like not enough fibrin.]

Visual Clot – First Contact

Field notes after Visual Clot study participation:

**Participant number**	**(9)**
**Center**	[redacted]
**Gender**	[redacted]
**Job position**	[redacted]
**Anesthesia experience in years**	[redacted]
**Performed ROTEM per year**	[redacted]
**Self-rated ROTEM skills**	[redacted]

Question 1: What do you like about Visual Clot? E.g., particular strengths?

Question 2: What do you dislike about Visual Clot? E.g., potential problems, limitations?

Answer question 1:

[Shows how many factors influence coagulability.] [You don't have to think as much as with conventional ROTEM.] [Intuitive.] [Eases diagnostic work.]

Answer question 2:

[Blood dripping from the clot might mislead the user and make him give anticoagulants, even if the patient is not bleeding. Critical discussion or interpretation is bypassed.]

Visual Clot – First Contact

Field notes after Visual Clot study participation:

**Participant number**	**(10)**
**Center**	[redacted]
**Gender**	[redacted]
**Job position**	[redacted]
**Anesthesia experience in years**	[redacted]
**Performed ROTEM per year**	[redacted]
**Self-rated ROTEM skills**	[redacted]

Question 1: What do you like about Visual Clot? E.g., particular strengths?

Question 2: What do you dislike about Visual Clot? E.g., potential problems, limitations?

Answer question 1:

[The visual presentation is easier to understand] [or to read than the waveform-numeric presentation of the ROTEM.]

Answer question 2:

[You can't see the exact number. You can't "rely" on the results 100%. You can't be sure about the degree of the deviation.]

Visual Clot – First Contact

Field notes after Visual Clot study participation:

**Participant number**	**(11)**
**Center**	[redacted]
**Gender**	[redacted]
**Job position**	[redacted]
**Anesthesia experience in years**	[redacted]
**Performed ROTEM per year**	[redacted]
**Self-rated ROTEM skills**	[redacted]

Question 1: What do you like about Visual Clot? E.g., particular strengths?

Question 2: What do you dislike about Visual Clot? E.g., potential problems, limitations?

Answer question 1:

[Makes a clear statement.] [Reading and interpreting the results is beeing done by an algorithm.]

Answer question 2:

[Results at the limit to normal are not shown.]

What happens with mistakes in pipetting? Could the Visual Clot put a question mark when an "impossible result" is being detected?

Visual Clot – First Contact

Field notes after Visual Clot study participation:

**Participant number**	**(12)**
**Center**	[redacted]
**Gender**	[redacted]
**Job position**	[redacted]
**Anesthesia experience in years**	[redacted]
**Performed ROTEM per year**	[redacted]
**Self-rated ROTEM skills**	[redacted]

Question 1: What do you like about Visual Clot? E.g., particular strengths?

Question 2: What do you dislike about Visual Clot? E.g., potential problems, limitations?

Answer question 1:

[Quick] and [easy] to interpret.

Answer question 2:

[No exact values / numbers. The exact degree of the disturbance is not know or can't even be estimated.]

Visual Clot – First Contact

Field notes after Visual Clot study participation:

**Participant number**	**(13)**
**Center**	[redacted]
**Gender**	[redacted]
**Job position**	[redacted]
**Anesthesia experience in years**	[redacted]
**Performed ROTEM per year**	[redacted]
**Self-rated ROTEM skills**	[redacted]

Question 1: What do you like about Visual Clot? E.g., particular strengths?

Question 2: What do you dislike about Visual Clot? E.g., potential problems, limitations?

Answer question 1:

[Fast learning.] [Easy, specially when not experienced in conventional ROTEM.] [Good overview.]

Answer question 2:

None.

Visual Clot – First Contact

Field notes after Visual Clot study participation:

**Participant number**	**(14)**
**Center**	[redacted]
**Gender**	[redacted]
**Job position**	[redacted]
**Anesthesia experience in years**	[redacted]
**Performed ROTEM per year**	[redacted]
**Self-rated ROTEM skills**	[redacted]

Question 1: What do you <u>like</u> about Visual Clot? E.g., particular strengths?

Question 2: What do you <u>dislike</u> about Visual Clot? E.g., potential problems, limitations?

Answer question 1:

[Well arranged.] [Quicker.]

Answer question 2:

[No quantitative data or information.]

Visual Clot – First Contact

Field notes after Visual Clot study participation:

**Participant number**	**(15)**
**Center**	[redacted]
**Gender**	[redacted]
**Job position**	[redacted]
**Anesthesia experience in years**	[redacted]
**Performed ROTEM per year**	[redacted]
**Self-rated ROTEM skills**	[redacted]

Question 1: What do you <u>like</u> about Visual Clot? E.g., particular strengths?

Question 2: What do you <u>dislike</u> about Visual Clot? E.g., potential problems, limitations?

Answer question 1:

[Intuitive kind of presentation.] [Even if long ago since last ROTEM interpretation,] it is [easy] and [quick] to use.

Answer question 2:

[Pac-Man and factors should be shown in different colors.]

Visual Clot – First Contact

Field notes after Visual Clot study participation:

**Participant number**	**(16)**
**Center**	[redacted]
**Gender**	[redacted]
**Job position**	[redacted]
**Anesthesia experience in years**	[redacted]
**Performed ROTEM per year**	[redacted]
**Self-rated ROTEM skills**	[redacted]

Question 1: What do you like about Visual Clot? E.g., particular strengths?

Question 2: What do you dislike about Visual Clot? E.g., potential problems, limitations?

Answer question 1:

[Until seeing the Visual Clot (VC), I couldn't imagine,] [that it could be presented in such a simple way!]

[If you see both at a time (Visual Clot and ROTEM), you would probably have a first look at the VC and then a second and more detailed look at the classic ROTEM.]

Answer question 2:

[I'm not sure if this simple presentation is enough. Don't we need more detailed information in order to be able to make a sound standing, good decision? For instance, how much fibrinogen (2gr. or 4gr.?) do I need to give?]

Visual Clot – First Contact

Field notes after Visual Clot study participation:

**Participant number**	**(17)**
**Center**	[redacted]
**Gender**	[redacted]
**Job position**	[redacted]
**Anesthesia experience in years**	[redacted]
**Performed ROTEM per year**	[redacted]
**Self-rated ROTEM skills**	[redacted]

Question 1: What do you like about Visual Clot? E.g., particular strengths?

Question 2: What do you dislike about Visual Clot? E.g., potential problems, limitations?

Answer question 1:

[Easy to understand form of presentation.] [Well structured.] [Clearly recognizable.] [Easier to read, because there are no numbers to interprete and which you might have to look up.]

Colors are fine that way. The similar colors of factors and fibrin do not interfere with each other.

Answer question 2:

[Movement of the clot is unnecessary and disturbing.

I feel there are not enough thrombocytes in the normal clot.]

Visual Clot – First Contact

Field notes after Visual Clot study participation:

**Participant number**	**(18)**
**Center**	[redacted]
**Gender**	[redacted]
**Job position**	[redacted]
**Anesthesia experience in years**	[redacted]
**Performed ROTEM per year**	[redacted]
**Self-rated ROTEM skills**	[redacted]

Question 1: What do you like about Visual Clot? E.g., particular strengths?

Question 2: What do you dislike about Visual Clot? E.g., potential problems, limitations?

Answer question 1:

[Simple presentation.]

Answer question 2:

If there are multiple factors missing, you need to look closely.

[The diagnoses are then very close together, it becomes difficult to differentiate two diagnoses spatially.]

Visual Clot – First Contact

Field notes after Visual Clot study participation:

**Participant number**	**(19)**
**Center**	[redacted]
**Gender**	[redacted]
**Job position**	[redacted]
**Anesthesia experience in years**	[redacted]
**Performed ROTEM per year**	[redacted]
**Self-rated ROTEM skills**	[redacted]

Question 1: What do you like about Visual Clot? E.g., particular strengths?

Question 2: What do you dislike about Visual Clot? E.g., potential problems, limitations?

Answer question 1:

Good! Clear statement. [Clear visualization.] [You can see immediately where the problem is.] [The doctor doesn't need to know and memorize the cutoff values.] The situation is presented in a black-and-white manner. [Simplicity creates clarity in emergency situations.]

Answer question 2:

[The visualization generates a qualitative statement. I don't know the algorithms, but it should be clear which cutoff values have been chosen.]

Visual Clot – First Contact

Field notes after Visual Clot study participation:

**Participant number**	**(20)**
**Center**	[redacted]
**Gender**	[redacted]
**Job position**	[redacted]
**Anesthesia experience in years**	[redacted]
**Performed ROTEM per year**	[redacted]
**Self-rated ROTEM skills**	[redacted]

Question 1: What do you like about Visual Clot? E.g., particular strengths?

Question 2: What do you dislike about Visual Clot? E.g., potential problems, limitations?

Answer question 1:

Easy to understand.

Answer question 2:

[You can’t really see the Pac-Man figure (in the hyperfibrinolysis scenario).]

Visual Clot – First Contact

Field notes after Visual Clot study participation:

**Participant number**	**(21)**
**Center**	[redacted]
**Gender**	[redacted]
**Job position**	[redacted]
**Anesthesia experience in years**	[redacted]
**Performed ROTEM per year**	[redacted]
**Self-rated ROTEM skills**	[redacted]

Question 1: What do you <u>like</u> about Visual Clot? E.g., particular strengths?

Question 2: What do you <u>dislike</u> about Visual Clot? E.g., potential problems, limitations?

Answer question 1:

[Very plausible!][Understandable / readable also in a stress situation.] [You don't need to know the cutoff values.]

Answer question 2:

[Maybe more complex situations cannot be presented sufficiently in this manner.]

Visual Clot – First Contact

Field notes after Visual Clot study participation:

**Participant number**	**(22)**
**Center**	[redacted]
**Gender**	[redacted]
**Job position**	[redacted]
**Anesthesia experience in years**	[redacted]
**Performed ROTEM per year**	[redacted]
**Self-rated ROTEM skills**	[redacted]

Question 1: What do you like about Visual Clot? E.g., particular strengths?

Question 2: What do you dislike about Visual Clot? E.g., potential problems, limitations?

Answer question 1:

[Good idea!] [Easy to learn!]

Answer question 2:

[Missing fibrin is not detected and understood easily.]

Visual Clot – First Contact

Field notes after Visual Clot study participation:

**Participant number**	**(23)**
**Center**	[redacted]
**Gender**	[redacted]
**Job position**	[redacted]
**Anesthesia experience in years in years**	[redacted]
**Performed ROTEM per year**	[redacted]
**Self-rated ROTEM skills**	[redacted]

Question 1: What do you <u>like</u> about Visual Clot? E.g., particular strengths?

Question 2: What do you <u>dislike</u> about Visual Clot? E.g., potential problems, limitations?

Answer question 1:

[Simple,][good.]

Answer question 2:

[The presentation of hyperfibrinogenemia confuses. Leave it away.]

Visual Clot – First Contact

Field notes after Visual Clot study participation:

**Participant number**	**(24)**
**Center**	[redacted]
**Gender**	[redacted]
**Job position**	[redacted]
**Anesthesia experience in years**	[redacted]
**Performed ROTEM per year**	[redacted]
**Self-rated ROTEM skills**	[redacted]

Question 1: What do you like about Visual Clot? E.g., particular strengths?

Question 2: What do you dislike about Visual Clot? E.g., potential problems, limitations?

Answer question 1:

[Through missing items,] [you quickly realize what the problem is.]

Answer question 2:

[The amount of missing factors or fibrinogen is not shown.]

[Hyperfibrinolysis and fibrin-missing are difficult to differenciate.]

Visual Clot – First Contact

Field notes after Visual Clot study participation:

**Participant number**	**(25)**
**Center**	[redacted]
**Gender**	[redacted]
**Job position**	[redacted]
**Anesthesia experience in years**	[redacted]
**Performed ROTEM per year**	[redacted]
**Self-rated ROTEM skills**	[redacted]

Question 1: What do you like about Visual Clot? E.g., particular strengths?

Question 2: What do you dislike about Visual Clot? E.g., potential problems, limitations?

Answer question 1:

[Foolproof!] [After only a short educational video you are already able to use it correctly!] [This is true for nurses, too. Can be used safely by all educational levels.] [It's timesaving.]

Answer question 2:

Maybe you don't really get involved anymore in understanding the process «rotational thrombelastometry», how it works and what's actually being measured. Might make you lazy. The consequences for the patient aren't negative, but for the doctor.

Visual Clot – First Contact

Field notes after Visual Clot study participation:

**Participant number**	**(26)**
**Center**	[redacted]
**Gender**	[redacted]
**Job position**	[redacted]
**Anesthesia experience in years**	[redacted]
**Performed ROTEM per year**	[redacted]
**Self-rated ROTEM skills**	[redacted]

Question 1: What do you like about Visual Clot? E.g., particular strengths?

Question 2: What do you dislike about Visual Clot? E.g., potential problems, limitations?

Answer question 1:

[Helps to find the problem more easily.] [With conventional ROTEM you need to know the (cutoff) values. And if you don't know the values and the combination algorithms, you need to get the guide book out of the drawer. [That's much easier and faster with the Visual Clot.]

Answer question 2:

[There's no graduation. Either all platelets have vanished, or they're all here.

Maybe the visual clot should change in size in order to show different grades of the coagulation deficit. Or only half of the platelets are missing.]

Visual Clot – First Contact

Field notes after Visual Clot study participation:

**Participant number**	**(27)**
**Center**	[redacted]
**Gender**	[redacted]
**Job position**	[redacted]
**Anesthesia experience in years**	[redacted]
**Performed ROTEM per year**	[redacted]
**Self-rated ROTEM skills**	[redacted]

Question 1: What do you like about Visual Clot? E.g., particular strengths?

Question 2: What do you dislike about Visual Clot? E.g., potential problems, limitations?

Answer question 1:

[The visualization is good and simple.] [Less items, with distinct assignment.] [You understand the animation intuitively.] [Facilitates the work of the user. With the conventional ROTEM a significant brainwork is needed.]

Answer question 2:

[The Heparineffect is a little tricky to interprete.]

Visual Clot – First Contact

Field notes after Visual Clot study participation:

**Participant number**	**(28)**
**Center**	[redacted]
**Gender**	[redacted]
**Job position**	[redacted]
**Anesthesia experience in years**	[redacted]
**Performed ROTEM per year**	[redacted]
**Self-rated ROTEM skills**	[redacted]

Question 1: What do you <u>like</u> about Visual Clot? E.g., particular strengths?

Question 2: What do you <u>dislike</u> about Visual Clot? E.g., potential problems, limitations?

Answer question 1:

[Much easier to capture than with the conventional ROTEM.] [Less brainwork needed.][Even without medical knowledge.] Reminds me of the picture riddles "find the ten differencies".

Answer question 2:

Whobbly clot confusing. PAC Man Trademark.

[And how much of the factor concentrate do I have to give now? The Visual Clot only tells me what's missing, but not how much.]

Visual Clot – First Contact

Field notes after Visual Clot study participation:

**Participant number**	**(29)**
**Center**	[redacted]
**Gender**	[redacted]
**Job position**	[redacted]
**Anesthesia experience in years**	[redacted]
**Performed ROTEM per year**	[redacted]
**Self-rated ROTEM skills**	[redacted]

Question 1: What do you like about Visual Clot? E.g., particular strengths?

Question 2: What do you dislike about Visual Clot? E.g., potential problems, limitations?

Answer question 1:

[Quick, simple to understand.] [You don't have to think long.]

Answer question 2:

[The dripping of the blood doesn't tell you if it's really bad or only marginally outside the normal. The Visual Clot is only black-and-white.]

Visual Clot – First Contact

Field notes after Visual Clot study participation:

**Participant number**	**(30)**
**Center**	[redacted]
**Gender**	[redacted]
**Job position**	[redacted]
**Anesthesia experience in years**	[redacted]
**Performed ROTEM per year**	[redacted]
**Self-rated ROTEM skills**	[redacted]

Question 1: What do you like about Visual Clot? E.g., particular strengths?

Question 2: What do you dislike about Visual Clot? E.g., potential problems, limitations?

Answer question 1:

[I liked it very much.] [I was much more relaxed when looking at the Visual Clot and interpreting the results. Perceived diagnostic confidence is higher] [You get the result / the answer much faster.] [You get an almost real picture of the bleeding, which is missing with the conventional ROTEM.]

Answer question 2:

It seems a little less academic. But it's about people's lives. Then it should be more important, as if it were academic.

Visual Clot – First Contact

Field notes after Visual Clot study participation:

**Participant number**	**(31)**
**Center**	[redacted]
**Gender**	[redacted]
**Job position**	[redacted]
**Anesthesia experience in years**	[redacted]
**Performed ROTEM per year**	[redacted]
**Self-rated ROTEM skills**	[redacted]

Question 1: What do you like about Visual Clot? E.g., particular strengths?

Question 2: What do you dislike about Visual Clot? E.g., potential problems, limitations?

Answer question 1:

[I intuitively understood.] [Felt safe.] [Easier to read than the ROTEM.]ROTEM curves are mostly contra-intuitiv.

Answer question 2:

None.

Visual Clot – First Contact

Field notes after Visual Clot study participation:

**Participant number**	**(32)**
**Center**	[redacted]
**Gender**	[redacted]
**Job position**	[redacted]
**Anesthesia experience in years**	[redacted]
**Performed ROTEM per year**	[redacted]
**Self-rated ROTEM skills**	[redacted]

Question 1: What do you like about Visual Clot? E.g., particular strengths?

Question 2: What do you dislike about Visual Clot? E.g., potential problems, limitations?

Answer question 1:

[Very good idea:] [Specially good, when not trained intensly and using it regularly.]

Answer question 2:

[Legend missing.] Not used to it.

Visual Clot – First Contact

Field notes after Visual Clot study participation:

**Participant number**	**(33)**
**Center**	[redacted]
**Gender**	[redacted]
**Job position**	[redacted]
**Anesthesia experience in years**	[redacted]
**Performed ROTEM per year**	[redacted]
**Self-rated ROTEM skills**	[redacted]

Question 1: What do you like about Visual Clot? E.g., particular strengths?

Question 2: What do you dislike about Visual Clot? E.g., potential problems, limitations?

Answer question 1:

[Easy. Selfexplaining.] [Clear picture.] [It doesn’t need the «translation work» from Curve to interpretation.]

Answer question 2:

[The picture «hypercoagubility» is confusing.]

Visual Clot – First Contact

Field notes after Visual Clot study participation:

**Participant number**	**(34)**
**Center**	[redacted]
**Gender**	[redacted]
**Job position**	[redacted]
**Anesthesia experience in years**	[redacted]
**Performed ROTEM per year**	[redacted]
**Self-rated ROTEM skills**	[redacted]

Question 1: What do you like about Visual Clot? E.g., particular strengths?

Question 2: What do you dislike about Visual Clot? E.g., potential problems, limitations?

Answer question 1:

[There's a good and clear visualization of what is missing.] [You feel safer.]

Answer question 2:

[The picture 'hypercoagubility' is confusing.]

Visual Clot – First Contact

Field notes after Visual Clot study participation:

Participant number	(35)
Center	[redacted]
Gender	[redacted]
Job position	[redacted]
Anesthesia experience in years	[redacted]
Performed ROTEM per year	[redacted]
Self-rated ROTEM skills	[redacted]

Question 1: What do you like about Visual Clot? E.g., particular strengths?

Question 2: What do you dislike about Visual Clot? E.g., potential problems, limitations?

Answer question 1:

[Easy.] [Fast.]

Answer question 2:

[You don't know the exact value, how far away from the cutoff you are.]

Visual Clot – First Contact

Field notes after Visual Clot study participation:

**Participant number**	**(36)**
**Center**	[redacted]
**Gender**	[redacted]
**Job position**	[redacted]
**Anesthesia experience in years**	[redacted]
**Performed ROTEM per year**	[redacted]
**Self-rated ROTEM skills**	[redacted]

Question 1: What do you like about Visual Clot? E.g., particular strengths?

Question 2: What do you dislike about Visual Clot? E.g., potential problems, limitations?

Answer question 1:

[Simple Interpretation.] Clear cutoff. [Fast decisionmaking possible.] [Very good, specially for beginners.]

Answer question 2:

ROTEM is not dichotomised. Repetitive measuring is not visualized.
