# Supplementary material for: Physicians’ Perceptions of a Situation Awareness–Oriented Visualization Technology for Viscoelastic Blood Coagulation Management (Visual Clot): Mixed Methods Study
Source: JMIR Serious Games. 2020 Dec 4;8(4):e19036. doi: 10.2196/19036 (PMC7748952; doi:10.2196/19036)
Supplement: Multimedia Appendix 3 [file games_v8i4e19036_app3.pdf]

Translated online survey announcement

Initial Mail

Subject: Visual Clot survey

Dear (enter name individually)

We hope you still remember our Visual Clot study, which we conducted almost a year ago. To be on the safe side we attach a picture of Visual Clot and of us in this mail. Meanwhile the main paper has been published in Anaesthesia (in the appendix). If you want to see your own performance, you were participant number (enter participant number individually).

We would like to ask you to fill out this extremely short survey. It will take at most one minute.

This would help us a lot in our further scientific work on this topic and product development.

Link to the survey -> https://de.surveymonkey.com/r/6J8MNPF

Thank you very much for your past and future help in advance.

Best wishes from Zurich,

Julian, David, Christoph, Sadiq and Tadzio.

Reminder

Subject: Reminder Visual Clot Survey

Dear (enter name individually)

We contact you regarding our Visual Clot survey. If you have already filled out the survey, we thank you very much. If you haven't yet, we have the link to the survey for you here. The survey will take a minute at most but please don't fill it out twice.

This would help us a lot in our further scientific work on this topic and product development.

Link to the survey -> https://de.surveymonkey.com/r/6J8MNPF

Thank you very much for your past and future help in advance.

Best regards,

Julian, David, Christoph, Sadiq and Tadzio.

PS: The current paper on the Visual Clot study is now available on PubMed. I have also added it again for you in the attachment.

Translated with www.DeepL.com/Translator (free version)
